# Licochalcone A Inhibits Melanoma Cell Survival by Suppressing HSP75 (TRAP1) and Disrupting Cellular Redox and Energy Homeostasis

**DOI:** 10.3390/ijms27209103

**Published:** 2026-10-14

**Authors:** Yoon-Jin Lee, Min Hyuk Choi, Ki Dam Kim, Sukh Que Park, Youin Bae, Yu Sung Choi, Sang Han Lee, Hae-Seon Nam, Moon Kyun Cho

**Affiliations:** 1 Division of Molecular Cancer Research, Soonchunhyang Medical Research Institute, Soonchunhyang University, Cheonan 31151, Republic of Korea; leeyj@sch.ac.kr (Y.-J.L.); m1037624@sch.ac.kr (S.H.L.); namhs@sch.ac.kr (H.-S.N.); 2 Department of Biochemistry, College of Medicine, Soonchunhyang University, Cheonan 31151, Republic of Korea; 3 Department of Dermatology, Soonchunhyang University Hospital, Seoul 04401, Republic of Korea; 146079@schmc.ac.kr (M.H.C.); 132351@schmc.ac.kr (K.D.K.); tempugene@naver.com (Y.B.); cardura@naver.com (Y.S.C.); 4 Department of Neurosurgery, Soonchunhyang University Hospital, Seoul 04401, Republic of Korea; drcolor@schmc.ac.kr; 5 Department of Tropical Medicine, College of Medicine, Soonchunhyang University, Cheonan 31151, Republic of Korea

**Keywords:** Licochalcone A, melanoma, HSP75, oxidative stress, apoptosis

## Abstract

Heat shock protein 75(HSP75), a mitochondrial chaperone of the HSP90 family, sustains tumor cell survival by restraining oxidative phosphorylation, favoring aerobic glycolysis, and limiting mitochondrial ROS to preserve redox homeostasis. Although Licochalcone A (LCA), a flavonoid isolated from licorice root, displays anticancer activity across diverse tumors, whether HSP75 suppression contributes to its anti-melanoma effects remains undefined. This study assessed HSP75 expression in melanoma and evaluated the anti-melanoma efficacy of LCA. Expression of HSP75 was compared between melanoma and normal skin specimens by Western blotting. G361 melanoma cells were exposed to LCA, after which HSP75 expression, cell viability (MTT and trypan blue assays), intracellular ROS, the GSH/GSSG balance, ATP-associated luminescence, AMPK phosphorylation, MMP2, and apoptosis-related proteins were examined. HSP75 was also silenced by siRNA in normal melanocytes (HEMn-MP) and G361 cells, alone or combined with LCA treatment, with HSP75, p-AMPK, MMP2, cleaved PARP, and cleaved caspase-3 assessed by Western blotting. Melanoma tissue showed elevated HSP75 relative to normal tissue. LCA reduced HSP75 expression and viability while raising ROS, shifting the GSH/GSSG ratio toward oxidative stress, and reducing ATP-associated luminescence. AMPK phosphorylation rose, MMP2 declined, and pro-apoptotic markers (Bax, cleaved caspase-3, cleaved PARP) increased. HSP75 knockdown alone reproduced the LCA-induced changes in ATP-associated luminescence, p-AMPK, cleaved PARP, cleaved caspase-3, and MMP2, and combined knockdown with LCA further enhanced these changes in G361 cells, which were largely attenuated in normal HEMn-MP melanocytes. LCA suppresses melanoma cell survival by inducing cellular oxidative stress, reducing ATP-associated luminescence, and promoting apoptosis, with HSP75 suppression contributing to the ATP/AMPK and apoptotic responses. These findings support further evaluation of LCA and HSP75 in melanoma.

## 1. Introduction

Melanoma, which arises from the malignant transformation of pigment-producing melanocytes, is the most lethal form of skin cancer despite accounting for only a small proportion of cutaneous malignancies [1,2]. Its pronounced capacity for early dermal invasion and rapid dissemination to regional lymph nodes and distant organs underlies the disproportionately high mortality associated with this tumor [3]. Although targeted inhibition of the MAPK pathway and immune checkpoint blockade have improved clinical outcomes in a subset of patients, intrinsic and acquired resistance frequently limit durable benefit, and advanced or metastatic melanoma continues to carry an unfavorable prognosis [4,5]. These limitations underscore the continued need to identify novel molecular vulnerabilities that can be therapeutically exploited in melanoma.

Metabolic reprogramming, characterized by enhanced aerobic glycolysis together with adaptive remodeling of mitochondrial oxidative phosphorylation (OXPHOS), is widely recognized as a hallmark of malignant transformation that supports tumor cell proliferation and survival under conditions of metabolic and oxidative stress [6,7]. Mitochondria occupy a central position in this adaptive process, coordinating ATP production, reactive oxygen species (ROS) generation, and the activation of apoptosis-associated signaling pathways [8,9]. Because excessive ROS accumulation and depletion of cellular energy reserves can trigger programmed cell death, cancer cells frequently rely on regulatory mechanisms that constrain mitochondrial respiratory activity and preserve redox homeostasis, thereby sustaining a pro-survival glycolytic phenotype [9,10,11].

Heat shock protein 75 (HSP75), also referred to as tumor necrosis factor receptor-associated protein 1 (TRAP1), is a mitochondrial matrix chaperone of the HSP90 family that plays a central role in this metabolic adaptation [12,13]. By restraining electron transport chain activity, HSP75 limits OXPHOS-derived ROS generation while favoring a glycolytic bioenergetic profile reminiscent of the Warburg phenotype, thereby protecting tumor cells against oxidative and metabolic insults [13,14]. Elevated HSP75 expression has been reported in several malignancies, including colorectal, prostate, and breast cancers, where it has been associated with enhanced cell survival and resistance to apoptosis [15,16,17]. Despite this growing recognition of HSP75 as a mitochondrial regulator of tumor survival, its expression pattern and functional contribution in human melanoma remain largely uncharacterized.

**Figure 1:**
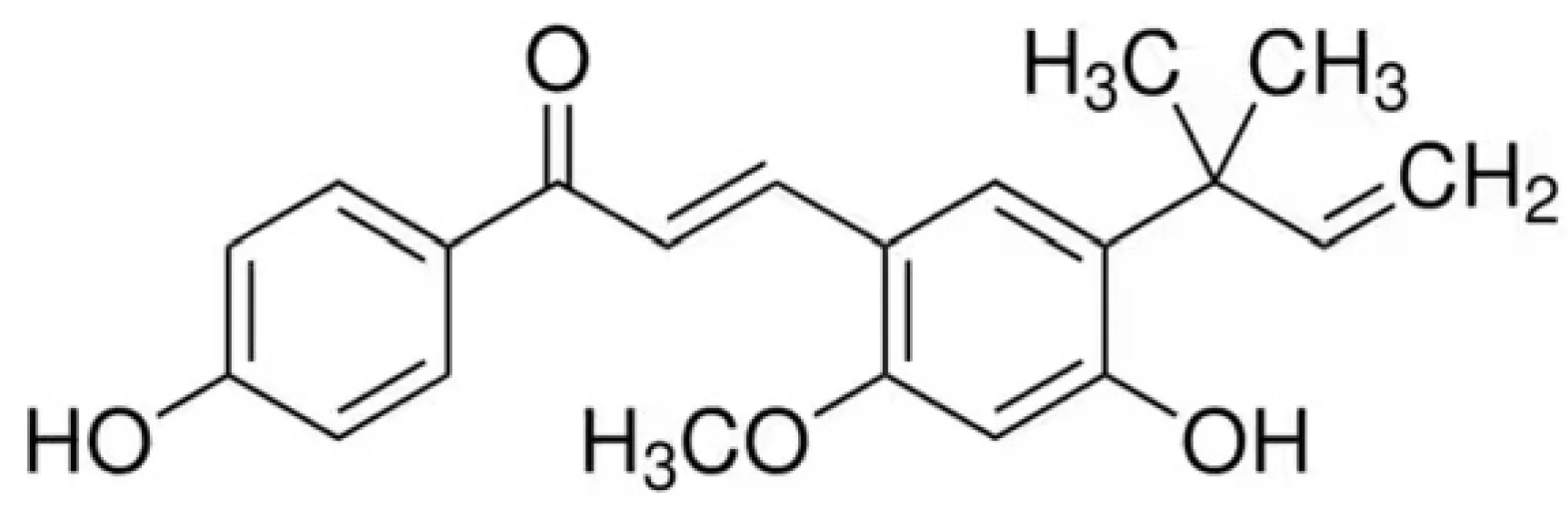
Chemical structure of the polyphenolic compound Licochalcone A (LCA) evaluated in this study.

Licochalcone A (LCA) is a chalcone-type flavonoid isolated from the roots of Glycyrrhiza (licorice) species (Figure 1) that has attracted considerable attention for its broad anticancer activity [18,19]. Previous studies indicate that LCA suppresses proliferation and induces apoptosis in various cancer cell types through mechanisms involving mitochondrial dysfunction and disruption of redox homeostasis [19,20]. Although these findings support the anticancer potential of LCA, whether its cytotoxic effects in melanoma are mechanistically linked to HSP75-dependent regulation of mitochondrial metabolism and redox balance has not previously been investigated.

Given that HSP75 sustains tumor cell survival by limiting OXPHOS-associated ROS production and preserving glycolytic flux, we hypothesized that HSP75 represents a mitochondrial vulnerability in melanoma that may contribute to the response to LCA. In the present study, we compared HSP75 expression between human melanoma and normal skin tissue and evaluated whether HSP75 suppression contributes to the cellular redox, energy-stress, and apoptotic responses induced by LCA in melanoma cells relative to normal melanocytes. These findings were intended to clarify the contribution of HSP75 to melanoma cell survival and its relationship to the anti-melanoma activity of LCA.

## 2. Results

### 2.1. Expression of HSP75 in Melanoma Tissues and Normal Skin Tissues

**Figure 2:**
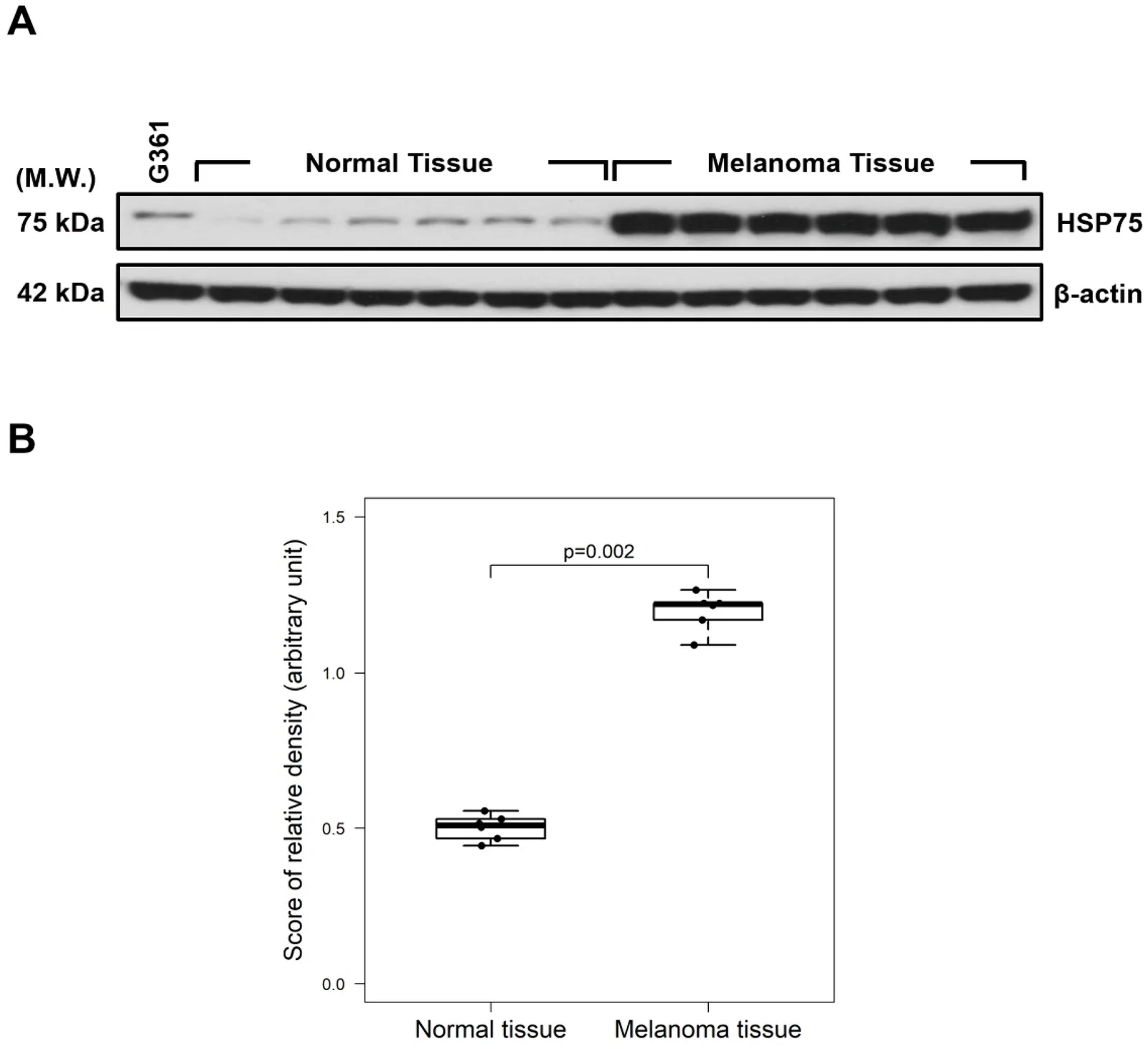
HSP75 protein expression in human normal skin and malignant melanoma tissues. (**A**) Western blot analysis. HSP75 levels were markedly increased in melanoma tissues compared to normal tissues. β-actin served as a loading control. (**B**) Relative HSP75 expression normalized to β-actin (arbitrary units). Box plots show the distribution of individual values, with each dot representing one tissue specimen (*n* = 6 per group). β-actin normalization controls for protein loading but does not correct for differences in melanocyte content. Statistical significance was assessed using the Mann–Whitney U test.

Western blot analysis was performed to assess the expression levels of HSP75 in melanoma tissues (*n* = 6) and normal skin tissues (*n* = 6). HSP75 expression was markedly increased in melanoma tissue samples relative to normal skin tissues (Figure 2A), demonstrating higher HSP75 abundance in the melanoma tissue lysates examined. HSP75 band intensities were normalized to β-actin as a loading control. The Mann–Whitney U test indicated a median (interquartile range) of 0.5091 (0.4762–0.5257) for normal skin tissues and 1.2203 (1.1819–1.2239) for melanoma tissues with respect to HSP75 expression, with a statistically significant difference (*p* = 0.002) between the two groups (Figure 2B). Because donor characteristics and melanoma stage were unavailable and no melanocyte-marker normalization was performed, this finding represents an exploratory comparison of whole-tissue lysates and does not establish increased HSP75 expression per melanoma cell.

### 2.2. Effects of LCA on Cell Viability in G361 and HEMn-MP Cells

**Figure 3:**
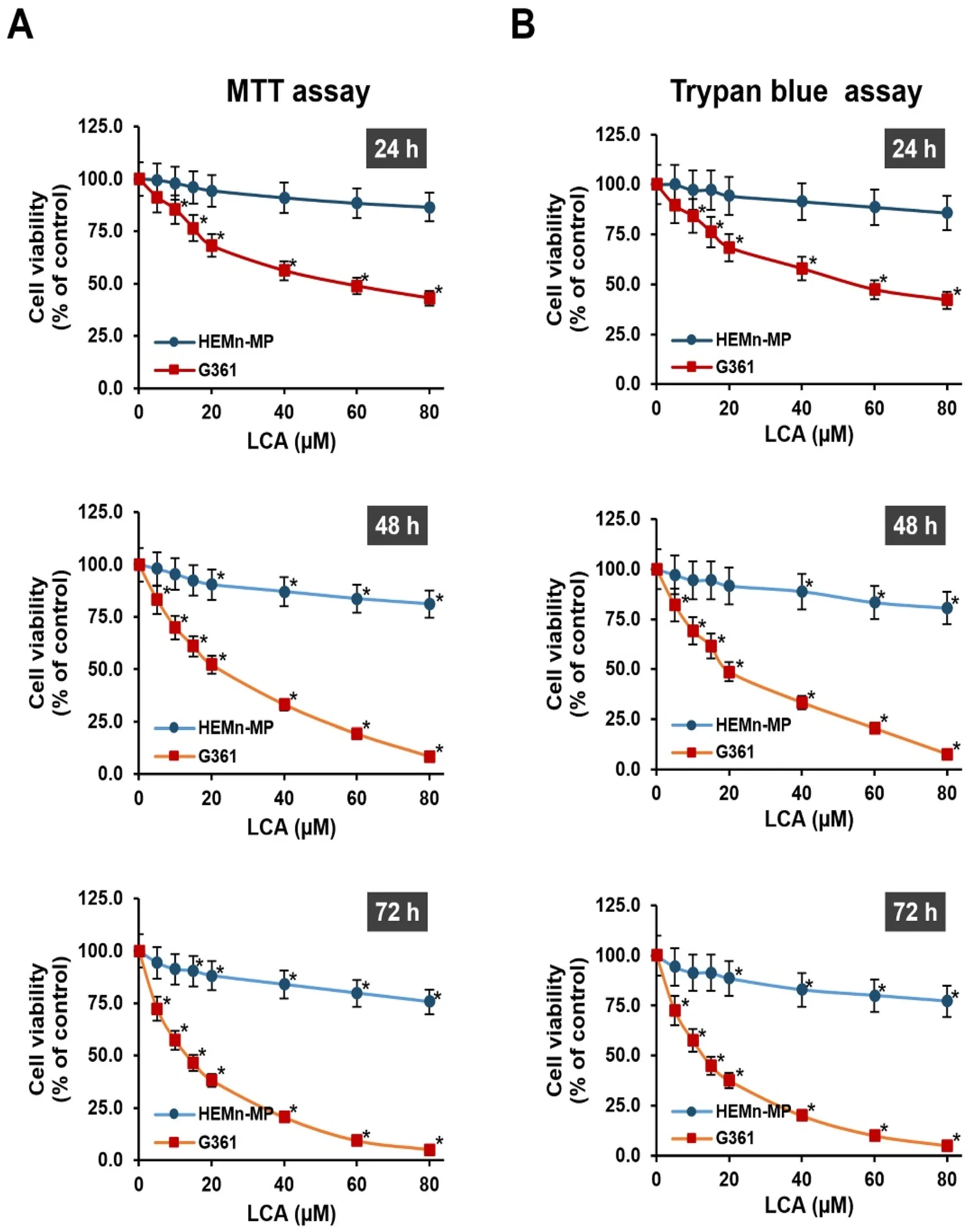
LCA induces a concentration- and time-dependent decrease in melanoma cell viability. (**A**) Viability of HEMn-MP and G361 melanoma cells following treatment with increasing concentrations of LCA for 24, 48, and 72 h, assessed using the MTT assay. (**B**) Cell viability under identical treatment conditions, assessed using the trypan blue exclusion assay. Data are presented as the mean ± SD (*n* = 3 independent experiments). Statistical significance was assessed by one-way ANOVA with Tukey’s post hoc test, performed separately for each cell line at each time point. Only comparisons with the untreated control (0 μM) are indicated (* *p* < 0.05).

To evaluate the cytotoxic effects of LCA on melanoma cells, G361 melanoma cells and HEMn-MP normal melanocytes were treated with increasing concentrations of LCA for 24, 48, and 72 h, and cell viability was assessed using the MTT assay (Figure 3A). At all three time points, LCA induced a concentration-dependent decrease in G361 cell viability, whereas HEMn-MP cells showed only minimal changes across the same concentration range. The reduction in G361 viability became more pronounced with longer exposure, with the most substantial decreases observed at 72 h, while HEMn-MP viability remained largely preserved regardless of treatment duration. Based on these MTT data, the IC_50_ values of LCA in G361 cells were 55.3 ± 1.9, 20.3 ± 0.6, and 12.5 ± 0.5 μM at 24, 48, and 72 h, respectively. In contrast, HEMn-MP viability remained above 75% even at the highest concentration tested (80 μM); therefore, the IC_50_ in HEMn-MP cells could not be reliably determined and was considered to exceed 80 μM at all time points. Because the HEMn-MP IC_50_ was not reached within the tested concentration range, exact selectivity index (SI) values could not be calculated. Using >80 μM as the lower bound for the HEMn-MP IC_50_, the corresponding minimum SI estimates (IC_50_ of HEMn-MP/IC_50_ of G361) were >1.4, >3.9, and >6.4 at 24, 48, and 72 h, respectively.

Consistent results were obtained using the trypan blue exclusion assay (Figure 3B), which similarly showed a concentration- and time-dependent reduction in G361 cell viability across the 24, 48, and 72 h time points, with minimal effect on HEMn-MP cells. Together, these two independent viability assays confirm that LCA selectively and progressively reduces melanoma cell viability in a concentration- and time-dependent manner while sparing normal melanocytes.

### 2.3. Effects of LCA Treatment on Oxidative Stress in G361 and HEMn-MP Cells

**Figure 4:**
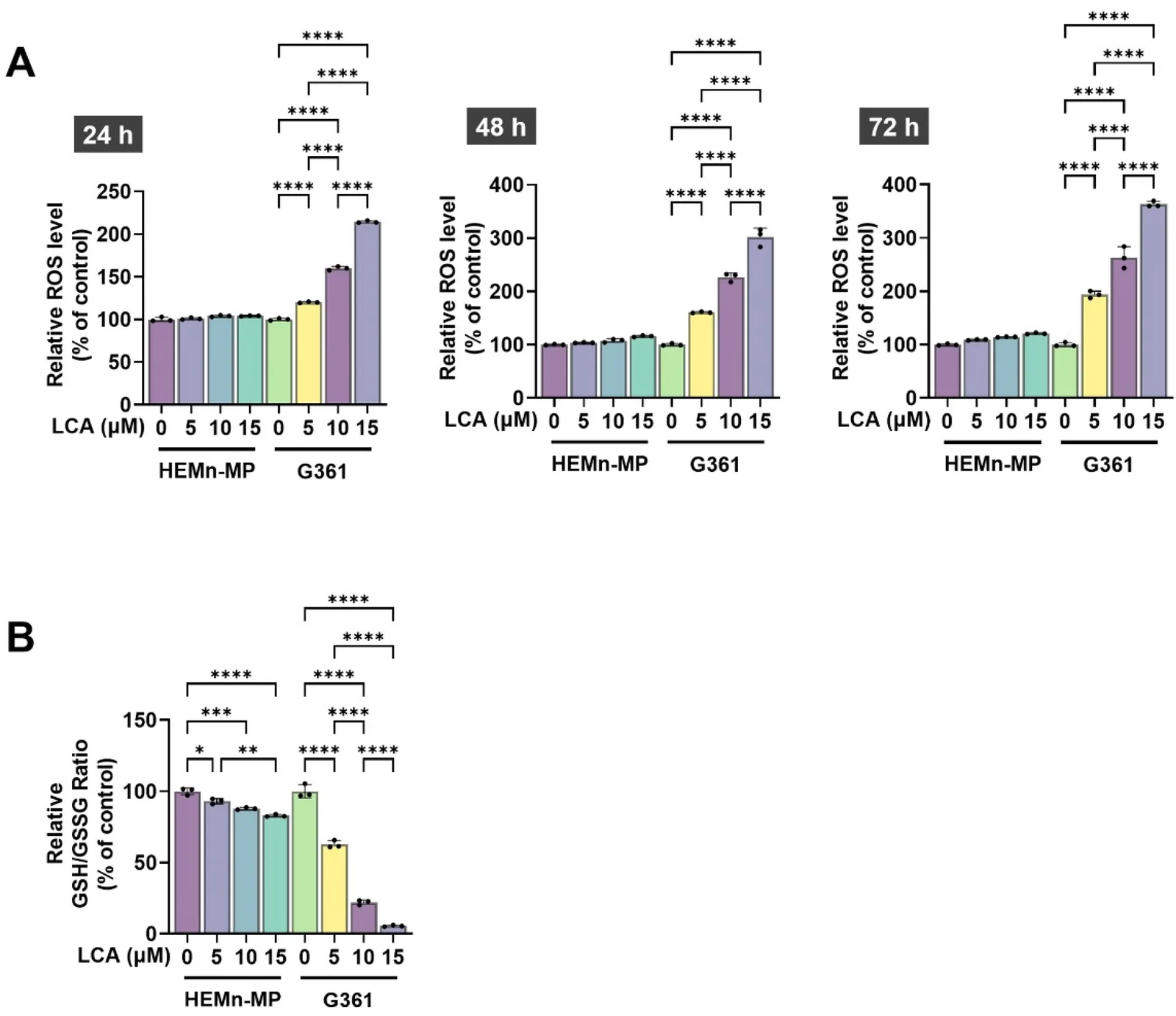
LCA induces oxidative stress and redox imbalance in melanoma cells. (**A**) Relative ROS levels in HEMn-MP and G361 cells following treatment with LCA (0, 5, 10, and 15 μM) for 24, 48, and 72 h, measured by DCFDA-derived DCF fluorescence, normalized to MTT viability, and expressed as percentage of control. (**B**) Relative GSH/GSSG ratio in HEMn-MP and G361 cells following treatment with LCA (0, 5, 10, and 15 μM) for 48 h, expressed as percentage of control. Data are presented as the mean ± SD (*n* = 3 independent experiments), with individual data points shown. Statistical significance was assessed by one-way ANOVA with Tukey’s post hoc test; brackets indicate pairwise comparisons between the groups shown (* *p* < 0.05; ** *p* < 0.01; *** *p* < 0.001; **** *p* < 0.0001).

To determine whether LCA-induced cytotoxicity was associated with oxidative stress, intracellular ROS levels were measured in HEMn-MP and G361 cells following treatment with increasing concentrations of LCA for 24, 48, and 72 h using DCFDA-derived DCF fluorescence (Figure 4A). LCA induced a concentration- and time-dependent increase in ROS levels in G361 cells, with the most pronounced elevation observed at the highest concentration and longest exposure duration, whereas ROS levels in HEMn-MP cells remained largely unchanged across all treatment conditions. These findings indicate that LCA selectively induces oxidative stress in melanoma cells.

To further characterize the impact of LCA on cellular redox homeostasis, the GSH/GSSG ratio was measured following LCA treatment (Figure 4B). LCA induced a concentration-dependent decrease in the GSH/GSSG ratio in G361 cells, whereas HEMn-MP cells exhibited only minimal changes. Taken together with the increased ROS levels, these results suggest that LCA disrupts redox homeostasis preferentially in melanoma cells.

### 2.4. LCA Induces Morphological Changes, Reduced ATP-Associated Luminescence, and Reduced Cell Growth Capacity in G361 and HEMn-MP Cells

**Figure 5:**
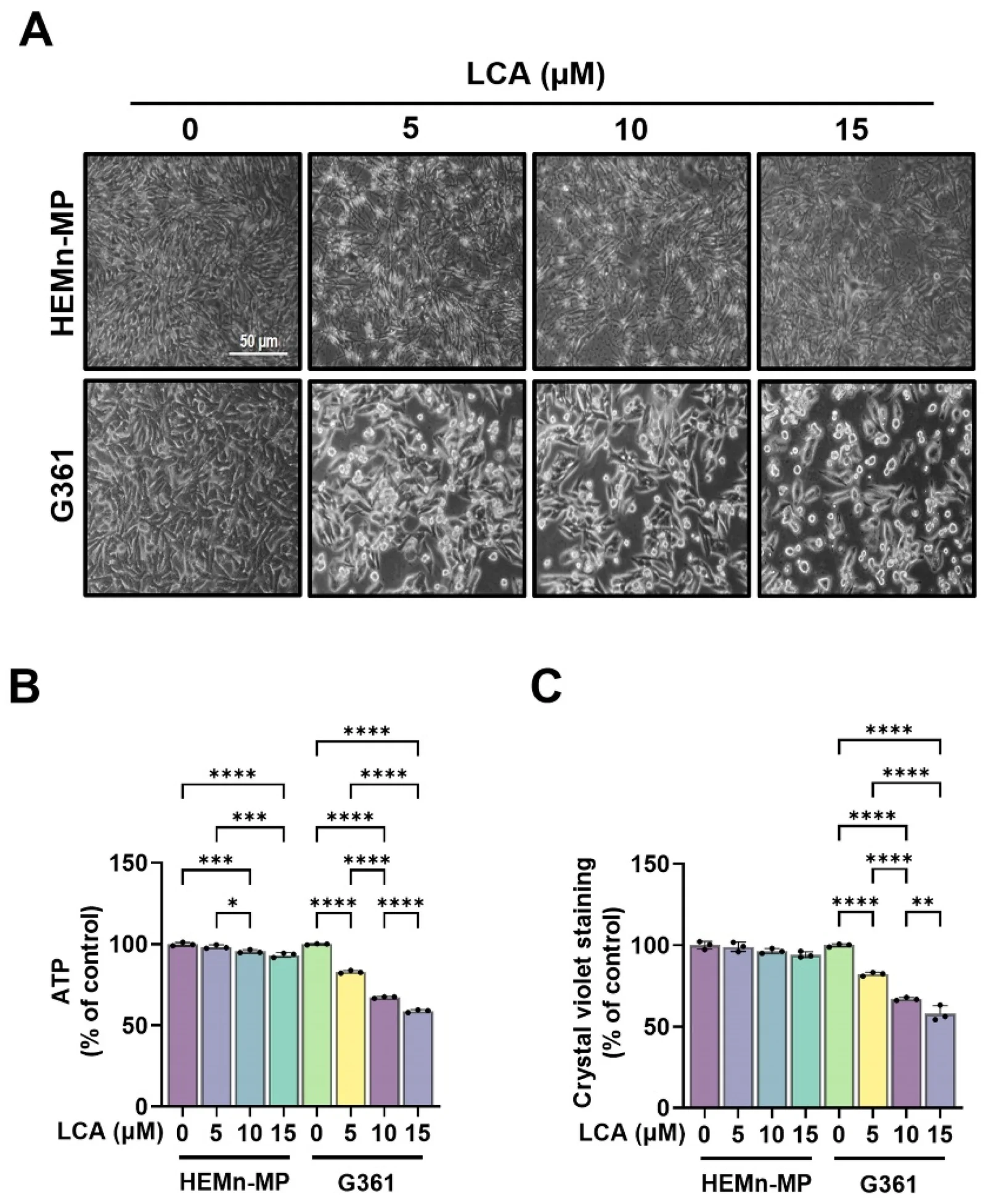
LCA induces morphological changes, reduced ATP-associated luminescence, and reduced growth capacity in G361 cells. (**A**) Representative phase-contrast images of HEMn-MP and G361 cells following treatment with LCA (0, 5, 10, and 15 μM) for 48 h. (**B**) ATP-associated luminescence in HEMn-MP and G361 cells following LCA treatment, expressed as percentage of control. (**C**) Cell growth capacity assessed by crystal violet staining following LCA treatment, expressed as percentage of control. Data in (**B**,**C**) are presented as the mean ± SD (*n* = 3 independent experiments), with individual data points shown. Statistical significance was assessed by one-way ANOVA with Tukey’s post hoc test; brackets indicate pairwise comparisons between the groups shown (* *p* < 0.05; ** *p* < 0.01; *** *p* < 0.001; **** *p* < 0.0001).

To further characterize the cytotoxic response to LCA, cell morphology, ATP-associated luminescence, and cell growth capacity were evaluated in HEMn-MP and G361 cells following treatment with increasing concentrations of LCA. Phase-contrast microscopy revealed that LCA treatment induced progressive morphological changes in G361 cells, including cell shrinkage, rounding, and detachment, in a concentration-dependent manner, whereas HEMn-MP cells retained their typical adherent morphology across all treatment conditions (Figure 5A). Consistent with these morphological alterations, ATP-associated luminescence was measured (Figure 5B). LCA induced a concentration-dependent decrease in ATP-associated luminescence in G361 cells, whereas HEMn-MP cells exhibited only minimal changes. Cell growth capacity was further evaluated using crystal violet staining (Figure 5C). LCA treatment produced a concentration-dependent reduction in crystal violet staining intensity in G361 cells, indicating impaired cell growth and survival capacity, whereas HEMn-MP cells showed comparatively little change across all treatment conditions. Taken together, these findings demonstrate that LCA selectively induces morphological deterioration, reduced ATP-associated luminescence, and suppression of growth capacity in G361 melanoma cells while sparing HEMn-MP cells.

### 2.5. LCA Modulates HSP75, AMPK Activation, Apoptosis-Related Proteins, and MMP2 Expression in G361 and HEMn-MP Cells

**Figure 6:**
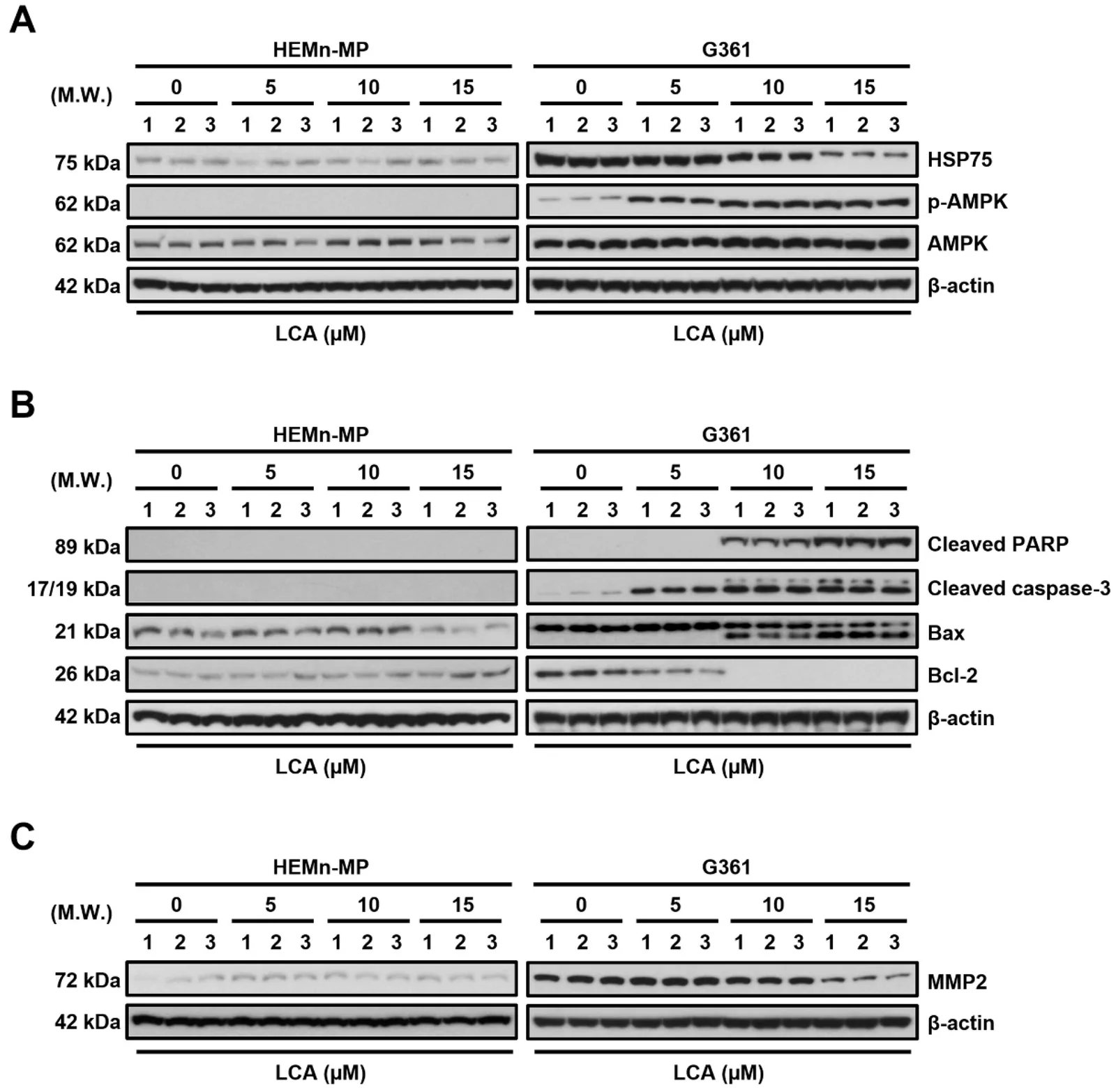

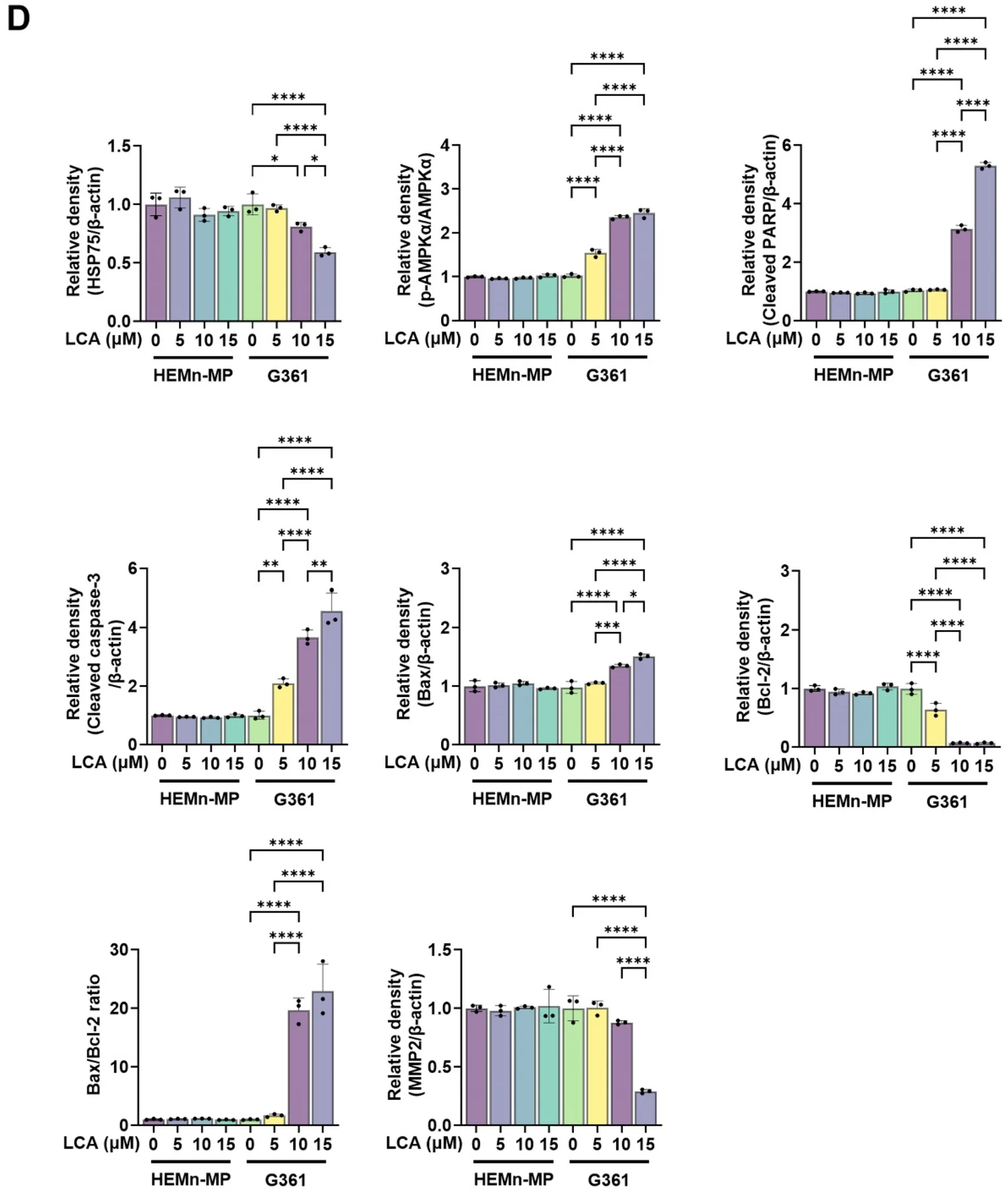
LCA modulates HSP75, AMPK, apoptosis-associated proteins, and MMP2 expression in HEMn-MP and G361 cells. (**A**) Representative Western blot images showing HSP75 expression and AMPK activation (p-AMPK, AMPK) in HEMn-MP and G361 cells treated with LCA (0, 5, 10, and 15 μM) for 48 h. (**B**) Expression of apoptosis-related proteins, including cleaved PARP, cleaved caspase-3, Bax, and Bcl-2, under the same treatment conditions. (**C**) MMP2 expression, with β-actin used as a loading control throughout. (**D**) Densitometric quantification of HSP75/β-actin, p-AMPK/AMPK, cleaved PARP/β-actin, cleaved caspase-3/β-actin, Bax/Bcl-2 ratio, and MMP2/β-actin. Molecular weight markers (kDa) are indicated to the left of each immunoblot. Data are presented as the mean ± SD (*n* = 3 independent experiments). Statistical significance was assessed by one-way ANOVA with Tukey’s post hoc test for multiple comparisons within each cell line. Brackets indicate pairwise comparisons * *p* < 0.05, ** *p* < 0.01, *** *p* < 0.001, **** *p* < 0.0001.

To elucidate the molecular mechanisms underlying the selective cytotoxicity of LCA toward melanoma cells, the expression levels of HSP75, AMPK activation, apoptosis-associated proteins, and MMP2 were examined by Western blotting with densitometric quantification (Figure 6A–D). HSP75 expression was markedly decreased in G361 cells following LCA treatment in a concentration-dependent manner, whereas HEMn-MP cells showed comparatively little change across all treatment conditions (Figure 6A), indicating selective suppression of HSP75 in melanoma cells. In parallel with the decrease in ATP-associated luminescence, LCA induced a concentration-dependent increase in AMPK phosphorylation (p-AMPK/AMPK) in G361 cells, while total AMPK levels remained unchanged; in contrast, p-AMPK levels in HEMn-MP cells were minimally affected (Figure 6A). Western blot analysis of apoptosis-associated proteins revealed that LCA treatment significantly increased the expression of cleaved PARP and cleaved caspase-3 in G361 cells, most prominently at 10 and 15 μM, whereas these markers remained largely unchanged in HEMn-MP cells (Figure 6B). Similarly, the Bax/Bcl-2 ratio was increased in G361 cells in a concentration-dependent manner, indicative of a shift toward pro-apoptotic signaling; in contrast, HEMn-MP cells exhibited minimal alterations in this ratio (Figure 6B). In parallel, MMP2 expression was decreased in G361 cells following LCA treatment in a concentration-dependent manner, whereas HEMn-MP cells showed comparatively little change (Figure 6C), documenting an additional LCA-associated molecular change in G361 cells. Because functional migration or invasion assays were not performed, this MMP2 decrease was not interpreted as direct evidence of reduced invasive behavior.

Densitometric analyses across all examined markers confirmed that LCA selectively suppressed HSP75 and MMP2 expression while concomitantly activating AMPK phosphorylation and pro-apoptotic signaling (increased cleaved PARP, cleaved caspase-3, and Bax/Bcl-2 ratio) in G361 cells, with minimal effects in HEMn-MP cells (Figure 6D). Collectively, these findings suggest that the melanoma-selective cytotoxicity of LCA is associated with coordinated suppression of HSP75, activation of AMPK-mediated stress signaling, induction of apoptosis, and downregulation of MMP2.

### 2.6. Loss-of-Function Analysis Supports a Role for HSP75 Suppression in the Anti-Melanoma Activity of LCA

**Figure 7:**
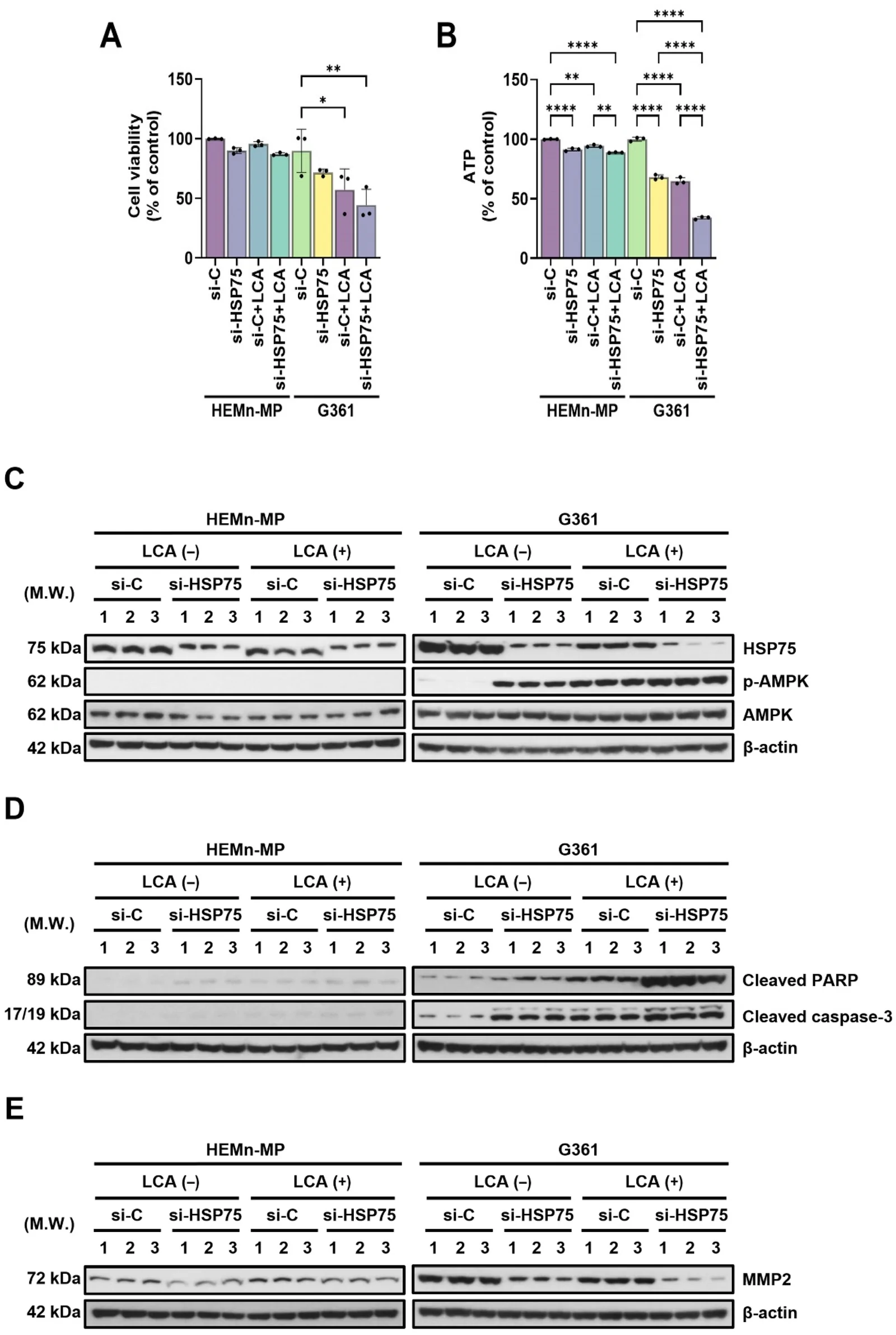

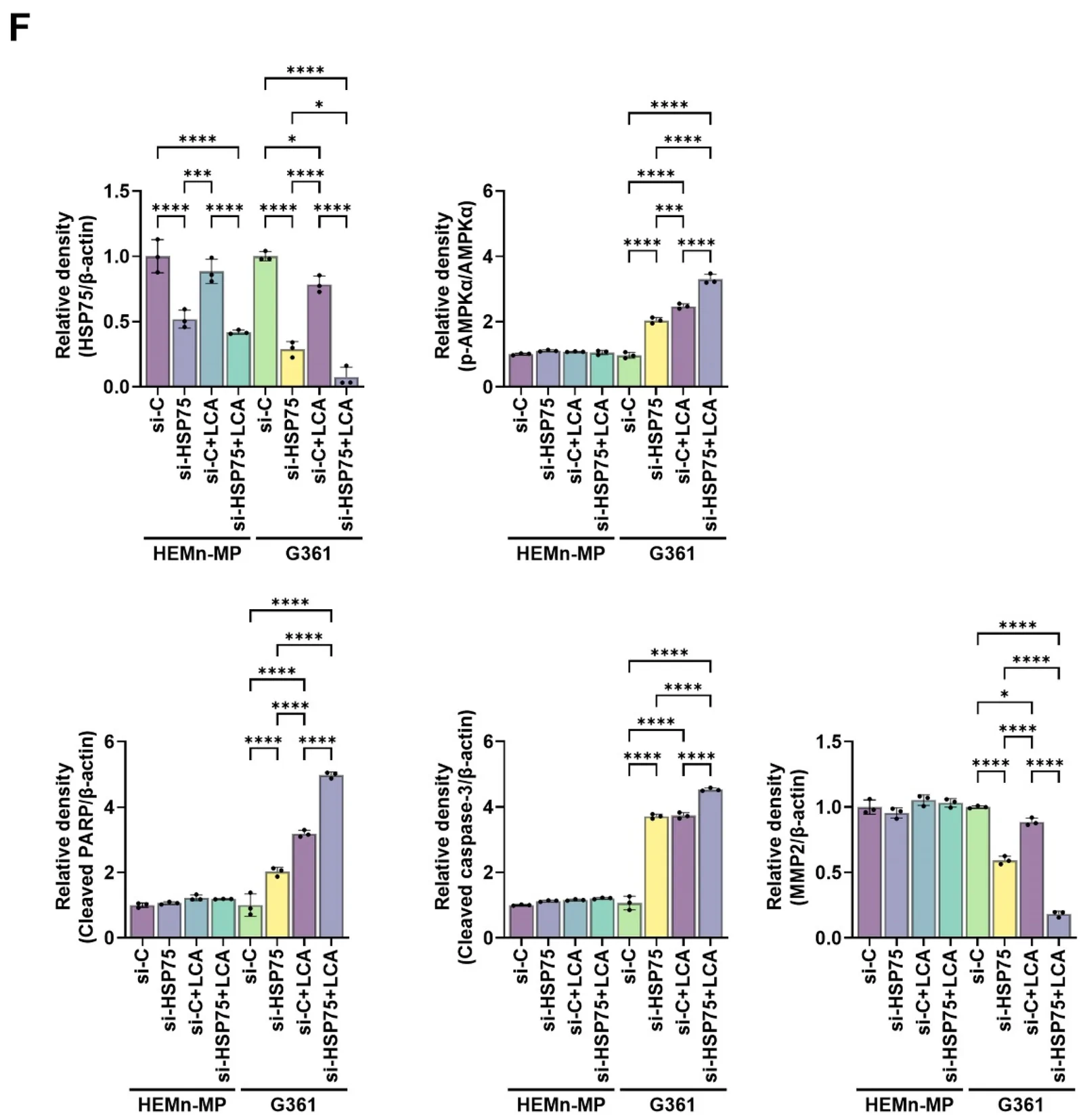
Genetic silencing of HSP75 mimics the effects of LCA on AMPK activation, apoptosis, and MMP2 expression, revealing an additive relationship between HSP75 knockdown and LCA treatment in G361 cells. Cells were transfected with control siRNA (si-C) or HSP75-targeting siRNA (si-HSP75) for 24 h, followed by treatment with vehicle or LCA 10 μM for 48 h, generating four experimental groups per cell line: si-C, si-HSP75, si-C + LCA, and si-HSP75 + LCA. (**A**) Cell viability was assessed by MTT assay. (**B**) ATP-associated luminescence was measured using a luminescence-based ATP assay. (**C**) Whole-cell lysates were subjected to Western blotting for HSP75, p-AMPK, and total AMPK, with β-actin as a loading control. (**D**) Western blotting for cleaved PARP and cleaved caspase-3, with β-actin as a loading control. (**E**) Western blotting for MMP2, with β-actin as a loading control. (**F**) Densitometric quantification of (**C**–**E**), presented as HSP75/β-actin, p-AMPK/AMPK, cleaved PARP/β-actin, cleaved caspase-3/β-actin, and MMP2/β-actin ratios, normalized to the si-C group. Data are expressed as mean ± SD from three independent experiments. Statistical significance among the four groups within each cell line was determined by one-way ANOVA followed by Tukey’s post hoc test. Brackets indicate pairwise comparisons: * *p* < 0.05, ** *p* < 0.01, *** *p* < 0.001, **** *p* < 0.0001.

To determine whether the anti-melanoma effects of LCA are mechanistically linked to HSP75 suppression, we performed a loss-of-function experiment using HSP75-targeting siRNA in combination with LCA in HEMn-MP and G361 cells. In G361 cells, cell viability was significantly reduced in the si-C + LCA and si-HSP75 + LCA groups compared with si-C, whereas si-HSP75 alone did not significantly differ from si-C; no significant difference was observed among the si-HSP75, si-C + LCA, and si-HSP75 + LCA groups. HEMn-MP cells showed no significant differences in viability across any of the four groups (Figure 7A). In G361 cells, si-HSP75 alone also significantly decreased ATP-associated luminescence compared with si-C, comparable to the decrease induced by LCA alone, and si-HSP75 + LCA further decreased this signal beyond both single treatments (Figure 7B).

Western blot analysis confirmed HSP75 knockdown in both cell lines (Figure 7C), and densitometry showed reduction to approximately 29% of si-C in G361 cells and 52% in HEMn-MP cells, indicating partial silencing with a substantial residual pool of the HSP75 protein in both cell lines (Figure 7F). In G361 cells, LCA further reduced HSP75 expression in si-HSP75-transfected cells (Figure 7F), and si-HSP75 alone significantly increased p-AMPK, cleaved caspase-3, and cleaved PARP and decreased MMP2 compared with si-C. Relative to LCA alone, si-HSP75 acted to a comparable extent on cleaved caspase-3, to a lesser extent on p-AMPK and cleaved PARP, and to a greater extent on MMP2 suppression. For all four markers, si-HSP75 + LCA produced a further significant change beyond either single treatment (Figure 7C–F).

In HEMn-MP cells, the only significant pairwise differences were in HSP75/β-actin, reflecting siRNA-mediated knockdown; LCA alone did not significantly alter HSP75 levels, and no significant differences were observed for p-AMPK/AMPK, cleaved PARP/β-actin, cleaved caspase-3/β-actin, or MMP2/β-actin (Figure 7F).

Collectively, these findings indicate that si-HSP75 alone reproduced the effects of LCA on ATP-associated luminescence, p-AMPK, cleaved PARP, cleaved caspase-3, and MMP2 in G361 cells, supporting HSP75 suppression as an important contributor to the anti-melanoma activity of LCA. Combined treatment with si-HSP75 and LCA further altered ATP-associated luminescence, p-AMPK, cleaved PARP, cleaved caspase-3, and MMP2 beyond either single treatment, whereas cell viability was not further affected. HEMn-MP cells showed minimal changes across all measures regardless of HSP75 or LCA status, consistent with the selective vulnerability of melanoma cells observed throughout this study.

## 3. Discussion

In the present study, we demonstrated that LCA exerts selective anti-melanoma activity in which suppression of HSP75 is an important contributor, leading to oxidative stress, energy stress, and apoptotic cell death. LCA treatment reduced HSP75 expression in G361 melanoma cells but not in HEMn-MP normal melanocytes, and this suppression was accompanied by increased ROS generation, a decreased GSH/GSSG ratio, reduced ATP-associated luminescence, and activation of AMPK signaling (p-AMPK) selectively in G361 cells. These upstream changes converged on mitochondria-dependent apoptosis, evidenced by increased cleaved PARP, cleaved caspase-3, and altered Bax/Bcl-2 ratio, as well as suppression of MMP2, a marker associated with invasive potential. siRNA-mediated knockdown of HSP75 alone reproduced the effects of LCA on ATP-associated luminescence, p-AMPK, cleaved PARP, cleaved caspase-3 and MMP2, and combined HSP75 knockdown with LCA further potentiated these changes in G361 cells (Figure 7).

Beyond its general role in sustaining Warburg-like metabolism [12,21], HSP75 inhibits succinate dehydrogenase (SDH, complex II), leading to succinate accumulation and pseudohypoxic stabilization of HIF1α, a mechanism required for HSP75-driven tumorigenicity in multiple cancer models [14]. The same SDH-inhibitory interaction limits ROS-dependent opening of the mitochondrial permeability transition pore, protecting tumor cells from oxidative death under nutrient-restricted conditions [11]. This role is not uniform across tissues, however: elevated HSP75 correlates with survival and apoptosis resistance in colorectal, prostate, and breast cancers [15,16,17] yet acts as a tumor suppressor in OXPHOS-dependent malignancies such as clear-cell renal carcinoma [13,21], indicating that its functional consequences depend on tissue metabolic context and warranting direct assessment in melanoma, as undertaken here.

LCA is broadly characterized as a licorice-derived chalconoid with anticancer activity across multiple tumor types, acting through the mitochondrial apoptotic pathway, ROS-dependent signaling, and modulation of PI3K/Akt/mTOR, MAPK, and JAK2/STAT3, with effects on cell cycle arrest, autophagy, and invasion in lung, liver, breast, and gastrointestinal cancers [18,19]. In melanoma specifically, prior studies of LCA reported apoptosis via Sp1 downregulation and death receptor activation, autophagy via the miR-142-3p/Rheb/mTOR axis, and suppressed migration through Akt inhibition [18], but none examined mitochondrial chaperone targets such as HSP75. The present study addresses this gap by linking LCA-induced anti-melanoma activity to HSP75 suppression.

The increased ROS and more oxidized GSH/GSSG ratio observed selectively in G361 cells are compatible with the known relationship between HSP75 and complex II regulation [11,14]. Because HSP75 can restrain electron leak at complex II [14], reduced HSP75 expression could plausibly contribute to oxidative stress. However, ROS and GSH/GSSG were not measured after HSP75 knockdown in the present study, so these data do not establish that HSP75 suppression caused the redox changes observed after LCA treatment. Since resting GSH/GSSG ratios typically exceed 100:1, a falling ratio reflects consumption of antioxidant buffering capacity rather than a fixed level of damage [22,23]. The decreased GSH/GSSG ratio is therefore interpreted as accompanying the LCA-induced increase in cellular oxidative stress, consistent with reports that redox-disrupting natural products achieve selective cytotoxicity in melanoma in a ROS-dependent, NAC-reversible manner [24].

The AMPK activation following HSP75 suppression is consistent with cellular energy stress rather than a separate ROS-dependent event. A fall in the ATP-to-ADP(AMP) ratio activates AMPK allosterically and enhances LKB1-mediated Thr172 phosphorylation [25,26], and direct inhibition of mitochondrial ATP production activates AMPK within minutes in proportion to the energetic deficit [27]. This is reinforced by the concordance between pharmacological and genetic data: si-HSP75 alone reproduced both the ATP decrease and p-AMPK increase seen with LCA, and si-HSP75 + LCA further potentiated both (Figure 7B,C,F), consistent with HSP75 suppression being an important contributor to LCA-induced AMPK activation. However, because ATP levels were measured at the cell-population level, the observed decrease may reflect both a reduction in viable cell number and altered cellular energy status and therefore does not by itself establish mitochondrial bioenergetic dysfunction.

Because silencing was partial, si-HSP75 cells retained approximately 29% of the HSP75 protein, providing a substantial residual pool on which LCA could act; the combination therefore achieved greater total suppression than either intervention alone. This is the most plausible explanation for the further changes in ATP, p-AMPK, cleaved PARP, cleaved caspase-3 and MMP2 observed with si-HSP75 + LCA. That said, LCA is a polyphenolic flavonoid and is unlikely to be entirely target-selective, so HSP75-independent contributions to its anti-melanoma activity cannot be excluded from the present data; we therefore interpret HSP75 suppression as an important contributor to the anti-melanoma activity of LCA.

Selective cytotoxicity of LCA toward malignant over normal cells has been consistently observed across cancer types: LCA induced apoptosis in non-small cell lung cancer cells at 10–15 µM while sparing normal human embryonic lung fibroblasts [28], with similar selectivity reported in gastric, hepatocellular, and cervical cancer models [29,30,31]. Our data in G361 and HEMn-MP cells are consistent with this pattern, supporting a selective cytotoxic effect on malignant melanocytes.

MMP2 is a matrix metalloproteinase associated with extracellular-matrix remodeling and melanoma invasion [32,33]. In the present study, MMP2 was included as a molecular marker and its expression decreased after LCA treatment and HSP75 knockdown. Prior studies have linked HSP75(TRAP1) suppression to reduced MMP2 and impaired migration via attenuated STAT3 signaling in esophageal carcinoma [34], TRAP1 knockdown to enhanced suppression of MMP2/MMP9 by shikonin via AKT/mTOR [35], and LCA to MMP2 downregulation in oral squamous cell carcinoma through PI3K/Akt inhibition [36]. AMPK activation has also been associated with reduced MMP2-dependent invasion in other cancer models [37]. These reports provide biological context for the MMP2 decrease observed here; however, because no functional migration, invasion, or MMP2 activity assay was performed, the present data do not establish that LCA reduced invasive behavior.

Although our findings support LCA’s therapeutic potential in melanoma, with HSP75 suppression as a contributing mechanism, several limitations should be addressed. First, the small sample size of clinical tissues (*n* = 6 per group) and exclusive use of the G361 cell line may limit broader applicability. In particular, G361 carries BRAF V600E, the most common driver mutation in cutaneous melanoma [38]. Because HSP75 has been reported to regulate BRAF synthesis and downstream ERK signaling in BRAF-mutant cancer cells [39], HSP75 suppression may also affect BRAF-driven signaling in this context, and the response to LCA may differ in melanoma cells with other driver mutations. With regard to the tissue analysis, because clinical information on the tissue donors, including sex, anatomical site, and melanoma stage, was not available, the comparability of the melanoma and normal skin groups could not be ensured, and potential confounding by these factors could not be assessed or adjusted for. Furthermore, HSP75 expression in tissue lysates was not normalized to a melanocyte marker such as MITF, S100, or Melan-A, so the higher HSP75 signal in melanoma tissue may partly reflect differences in melanocyte content as well as differences in HSP75 expression. β-actin normalization controls for protein loading but does not resolve this cellular-composition confound. The tissue results are therefore exploratory and cannot establish melanoma-cell-specific HSP75 upregulation or an association with clinical stage.

Second, the anti-melanoma efficacy of LCA was not verified in an in vivo model. Third, the oral bioavailability of LCA is reportedly low (3.3%) due to extensive first-pass metabolism [40], raising the question of whether effective concentrations can be achieved in vivo. Fourth, mitochondrial function, including mitochondrial ROS, membrane potential, and oxygen consumption, was not directly assessed.

**Figure 8:**
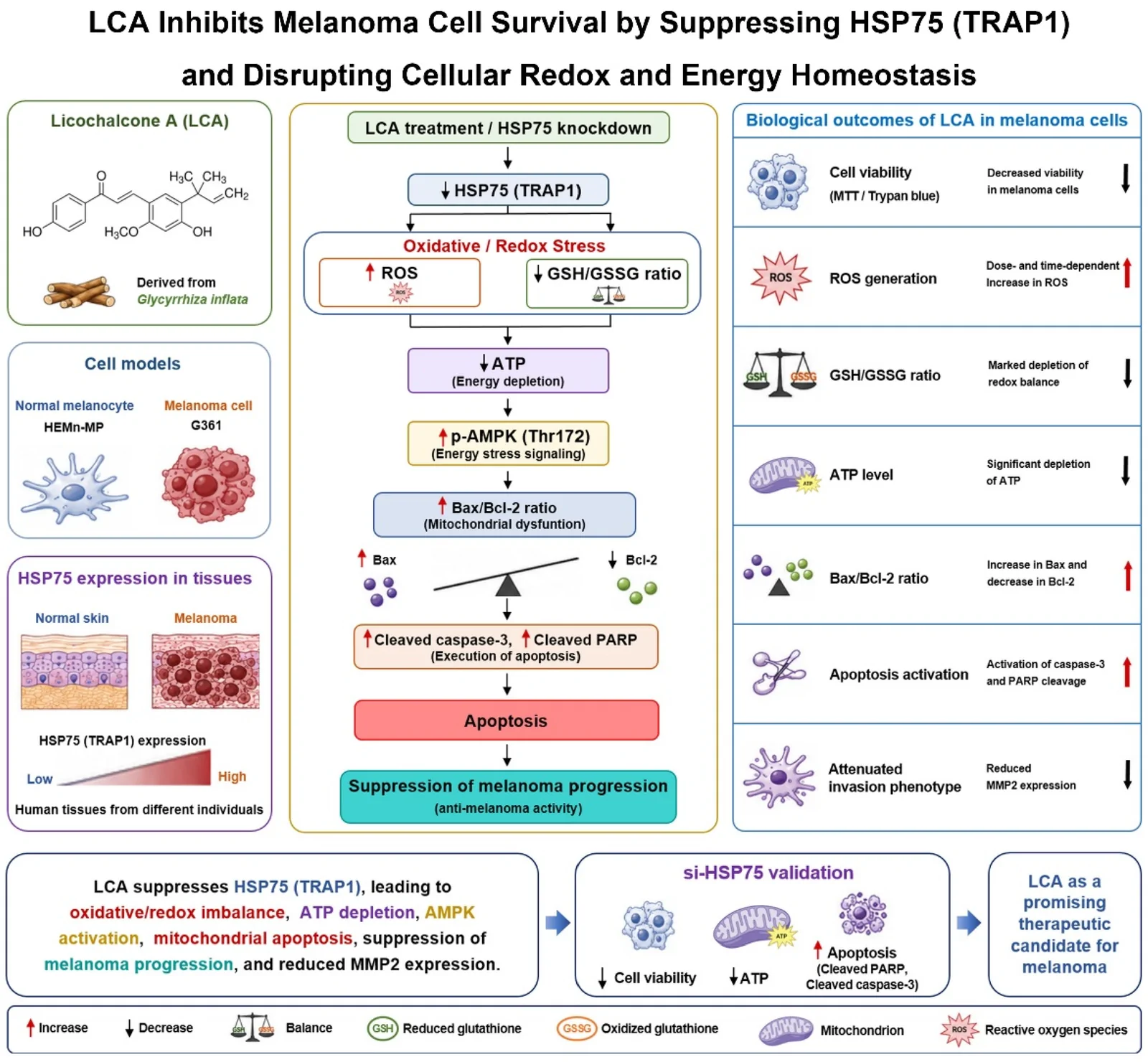
Proposed mechanism underlying the anti-melanoma effects of Licochalcone A (LCA) through suppression of HSP75. The schematic summarizes that LCA suppresses HSP75 and is accompanied by increased intracellular ROS and reduced ATP-associated luminescence. These changes are associated with activation of AMPK signaling (↑ p-AMPK) and apoptotic execution, as evidenced by increased cleavage of PARP and caspase-3 (↑ Cleaved PARP, ↑ Cleaved caspase-3). MMP2 expression is also reduced (↓ MMP2). Because ROS and GSH/GSSG were not measured after HSP75 knockdown and functional migration or invasion assays were not performed, the redox and MMP2 changes are presented as associated findings rather than direct evidence of HSP75-dependent redox causality or altered invasive behavior. These coordinated responses are consistent with reduced melanoma cell survival and increased apoptosis in G361 cells, while HEMn-MP cells remain largely unaffected. Created in BioRender. Lee, Y. J. (2026) https://BioRender.com/e7qbqj2.

Figure 8 summarizes the proposed mechanism, in which LCA-induced HSP75 suppression is accompanied by increased cellular oxidative stress, reduced ATP-associated luminescence, and AMPK activation, converging on intrinsic apoptosis. Because HSP75 normally stabilizes the mitochondrial permeability transition pore and restrains Bax-dependent outer membrane permeabilization [11,41], its loss releases this brake on apoptosis, consistent with the increased Bax/Bcl-2 ratio, cleaved PARP, and cleaved caspase-3 observed here (Figure 6), the latter two further potentiated by combined HSP75 knockdown and LCA treatment (Figure 7). This concordance suggests that HSP75 suppression contributes to caspase-dependent apoptotic execution, in addition to its association with cellular energy stress, while downregulation of MMP2 represents an additional molecular change associated with LCA treatment and HSP75 suppression; it is not interpreted here as direct evidence of altered invasive behavior. As these effects were selective to G361 cells and largely absent in HEMn-MP, HSP75 emerges as a mitochondrial node contributing to melanoma cell survival and apoptosis resistance.

## 4. Materials and Methods

### 4.1. Tissue Sample Collection and Preparation

A total of six normal skin tissues and six melanoma tissues were obtained from patients who underwent surgery between December 2015 and July 2017 in the Department of Plastic and Reconstructive Surgery of Soonchunhyang University Hospital Bucheon, Korea. The tissue samples used in this study were residual specimens from a previous study. The specimens were provided in a coded, de-identified form without personally identifiable information, and because they were obtained through secondary distribution, clinical information on the donors, including sex, anatomical site, and melanoma stage, was not available to the investigators. Consequently, matching by sex or anatomical site and analysis by clinical stage were not possible. The Institutional Review Board of Soonchunhyang University Hospital Seoul approved the secondary use of these samples (IRB No. 2018-04-005). The remaining specimens were frozen in liquid nitrogen immediately after resection and stored at −70 °C for Western blot analysis.

### 4.2. Reagents and Antibodies

Licochalcone A (LCA), dimethyl sulfoxide (DMSO), MTT reagent, crystal violet, and the β-actin antibody (A2228) were purchased from Sigma-Aldrich (St. Louis, MO, USA). LCA was dissolved in DMSO to prepare stock solutions. The anti-HSP75 (TRAP1) antibody (clone TR1, sc-13557) was obtained from Santa Cruz Biotechnology (Santa Cruz, CA, USA). The anti-MMP2 antibody (ab7032) was purchased from Abcam (Cambridge, UK). Primary antibodies against phospho-AMPKα (Thr172; 2535), AMPKα (2532), cleaved PARP (5625), cleaved caspase-3 (9664), Bax (2772), and Bcl-2 (4223), as well as horseradish peroxidase (HRP)-conjugated anti-rabbit IgG (7074) and anti-mouse IgG (7076) secondary antibodies, were obtained from Cell Signaling Technology (Danvers, MA, USA).

### 4.3. Cell Culture

Normal human epidermal melanocytes (HEMn-MP; Cascade Biologics, Portland, OR, USA) were cultured in Medium 254 supplemented with Human Melanocyte Growth Supplement according to the manufacturer’s instructions. The human malignant melanoma cell line G361 was obtained from the American Type Culture Collection (ATCC, Manassas, VA, USA) and maintained in Dulbecco’s Modified Eagle Medium (DMEM; WelGene, Gyeongsan, Republic of Korea) supplemented with 5% fetal bovine serum (Gibco, Gaithersburg, MD, USA), 100 U/mL penicillin, and 100 μg/mL streptomycin. All cells were maintained at 37 °C in a humidified incubator with 5% CO_2_.

### 4.4. Western Blot Analysis

Tissue samples and cultured cells were lysed using RIPA buffer supplemented with a protease inhibitor cocktail (Roche, Basel, Switzerland). Total protein concentrations were determined using the Bradford protein assay (Bio-Rad Laboratories, Hercules, CA, USA) according to the manufacturer’s instructions. Equal amounts of protein (50 μg per lane) were separated on 4–12% NuPAGE Bis-Tris gels (Invitrogen; Thermo Fisher Scientific, Waltham, MA, USA) and transferred onto polyvinylidene difluoride (PVDF) membranes (Cytiva, Amersham, UK). The membranes were blocked with 5% skim milk prepared in phosphate-buffered saline containing 0.05% Tween-20 (PBS-T) for 2 h at room temperature and then incubated overnight at 4 °C with the indicated primary antibodies (1:500, except for β-actin, which was used at 1:1000). After washing with PBS-T, the membranes were incubated with HRP-conjugated secondary antibodies (1:5000) for 2 h at room temperature. Immunoreactive bands were visualized using an enhanced chemiluminescence (ECL) detection kit (Cytiva, Amersham, UK). Band intensities were quantified using ImageJ software (version 1.54k; National Institutes of Health, Bethesda, MD, USA) and normalized to β-actin, except phospho-AMPK was normalized to total AMPK, and Bax was expressed relative to Bcl-2.

### 4.5. Cell Viability Assays

Cell viability was evaluated using MTT and trypan blue exclusion assays. G361 and HEMn-MP cells were seeded into 96-well plates at a density of 1 × 10^4^ cells/well and allowed to attach overnight. The cells were treated with LCA at concentrations of 0, 5, 10, 15, 20, 40, 60, and 80 μM for 24, 48, or 72 h. Following treatment, MTT solution (final concentration, 0.1 mg/mL) was added to each well and incubated for 3 h. The resulting formazan crystals were dissolved in DMSO, and absorbance was measured at 570 nm using a GloMax^®^-Multi Detection System (Promega, Madison, WI, USA).

For the trypan blue exclusion assay, cells were harvested after treatment, resuspended in phosphate-buffered saline (PBS), mixed with trypan blue solution, and counted using a hemocytometer. Cell viability was expressed as the percentage of viable cells relative to untreated control cells, which were defined as 100%. All experiments were performed in triplicate and repeated independently three times.

### 4.6. Determination of Intracellular Reactive Oxygen Species

Intracellular reactive oxygen species (ROS) levels were determined using the DCFDA/H_2_DCFDA Cellular ROS Assay Kit (ab113851, Abcam, Cambridge, UK). G361 and HEMn-MP cells were seeded into black-walled, clear-bottom 96-well plates and allowed to attach overnight before treatment with LCA (0, 5, 10, or 15 μM) for 24, 48, or 72 h. Following treatment, the culture medium was removed, and the cells were incubated with DCFDA working solution for 30 min at 37 °C in the dark. Fluorescence intensity was measured using a GloMax^®^-Multi Detection System (Promega, Madison, WI, USA) at excitation and emission wavelengths of 485 and 535 nm, respectively. DCF fluorescence in each treatment group was expressed relative to its corresponding control. The relative fluorescence value was then divided by the relative MTT viability measured under parallel treatment conditions and multiplied by 100. The resulting values are reported as MTT-normalized relative DCF fluorescence.

### 4.7. Determination of Intracellular Glutathione Redox Status

Intracellular glutathione redox status was assessed using the GSH/GSSG-Glo™ Assay (V6611; Promega, Madison, WI, USA) according to the manufacturer’s instructions. G361 and HEMn-MP cells were seeded into white opaque 96-well plates and treated with LCA at 0, 5, 10, or 15 μM for 48 h. Following treatment, intracellular reduced glutathione (GSH) and oxidized glutathione (GSSG) levels were measured independently using the corresponding detection reagents provided in the assay kit. Luminescence was recorded using a GloMax^®^-Multi Detection System (Promega, Madison, WI, USA). The intracellular GSH/GSSG ratio was subsequently calculated and normalized to the untreated control group as an indicator of cellular redox homeostasis.

### 4.8. Measurement of ATP-Associated Luminescence

ATP-associated luminescence was measured using the CellTiter-Glo^®^ Luminescent Cell Viability Assay (G7570; Promega, Madison, WI, USA) following the manufacturer’s protocol. G361 and HEMn-MP cells were seeded into white opaque 96-well plates and treated with LCA at 0, 5, 10, or 15 μM for 48 h. After treatment, an equal volume of CellTiter-Glo^®^ reagent was added directly to each well, and the plates were gently mixed on an orbital shaker for 2 min to induce complete cell lysis. The plates were then incubated at room temperature for 10 min to stabilize the luminescent signal. Luminescence was measured using a GloMax^®^-Multi Detection System (Promega, Madison, WI, USA), and the signal was expressed relative to that of the untreated control group. ATP and MTT assays were performed in separate experiments, without per-well normalization of the ATP-associated luminescence signal to cell number or total protein.

### 4.9. Crystal Violet Staining Assay

Long-term cell survival was additionally assessed using a crystal violet staining assay. G361 and HEMn-MP cells were seeded into 6-well plates and exposed to LCA at 0, 5, 10, or 15 μM for 48 h. Following treatment, the cells were fixed with absolute methanol for 15 min and stained with 0.5% crystal violet solution for 20 min at room temperature. Excess dye was removed by gently rinsing the plates with distilled water, after which the stained cells were allowed to air-dry completely. Representative images were captured using an inverted microscope. For quantitative analysis, the bound crystal violet was dissolved in 10% acetic acid, and absorbance was measured at 570 nm using a GloMax^®^-Multi Detection System (Promega, Madison, WI, USA).

### 4.10. Small Interfering RNA (siRNA) Transfection

To investigate the functional role of HSP75 in LCA-induced cellular responses, HSP75 expression was transiently silenced using small interfering RNA (siRNA). A Stealth RNAi™ siRNA targeting human HSP75 (TRAP1) (HSS110105; Invitrogen, Carlsbad, CA, USA) was used for gene silencing. G361 and HEMn-MP cells were seeded into 6-well or 96-well plates and transfected at approximately 40% confluence with either the HSP75-specific siRNA or a Stealth RNAi™ Negative Control duplex (12935-200; Invitrogen, Carlsbad, CA, USA) using Lipofectamine™ RNAiMAX Transfection Reagent (Invitrogen, Carlsbad, CA, USA) according to the manufacturer’s instructions. After 24 h of transfection, cells were treated with vehicle or LCA 10 μM for 48 h, generating four experimental groups per cell line: si-C, si-HSP75, si-C + LCA, and si-HSP75 + LCA. HSP75 knockdown was confirmed by Western blot analysis.

### 4.11. Statistical Analysis

Cell-culture experiments were independently performed three times, and the data are presented as the mean ± standard deviation (SD). Statistical analyses were conducted using SPSS software (version 17.0; IBM Corp., Armonk, NY, USA). The tissue analysis included six normal skin specimens and six melanoma specimens, with one quantified value per specimen. Differences between two groups were analyzed using the Mann–Whitney U test for non-parametric data. Comparisons among multiple groups were performed using one-way analysis of variance (ANOVA) followed by Tukey’s post hoc test. Quantitative analysis of Western blot band intensities was carried out using ImageJ software (version 1.54k, National Institutes of Health, Bethesda, MD, USA), and the resulting data were subjected to statistical analysis using SPSS. A value of *p* < 0.05 was considered statistically significant.

## 5. Conclusions

This study demonstrates that LCA selectively induces apoptotic cell death in G361 melanoma cells while largely sparing HEMn-MP normal melanocytes. LCA treatment was associated with HSP75 suppression, increased oxidative stress, reduced ATP-associated luminescence, and AMPK activation, together with an increased Bax/Bcl-2 ratio, cleaved PARP, and cleaved caspase-3, as well as reduced MMP2 expression. siRNA-mediated HSP75 knockdown reproduced the LCA-associated changes in ATP-associated luminescence, p-AMPK, apoptotic markers, and MMP2, supporting HSP75 suppression as an important contributor to the anti-melanoma action of LCA. Collectively, these findings support a contributory role for HSP75 suppression in the response to LCA and warrant further validation in additional melanoma models and with direct functional assays of mitochondrial and invasive phenotypes.

## Data Availability

The original contributions presented in this study are included in the article. Further inquiries can be directed to the corresponding author.

## Abbreviations

The following abbreviations are used in this manuscript:

AMPK	AMP-activated protein kinase
DMSO	Dimethyl sulfoxide
GSH/GSSG	Glutathione (reduced)/Glutathione disulfide (oxidized)
MMP2	Matrix metalloproteinase 2
MTT	3-(4,5-dimethylthiazol-2-yl)-2,5-diphenyltetrazolium bromide
NAC	N-acetylcysteine
PARP	Poly (ADP-ribose) polymerase
